# Associating an Entropy with Power-Law Frequency of Events

**DOI:** 10.3390/e20120940

**Published:** 2018-12-06

**Authors:** Evaldo M. F. Curado, Fernando D. Nobre, Angel Plastino

**Affiliations:** 1 Centro Brasileiro de Pesquisas Físicas and National Institute of Science and Technology for Complex Systems, Rua Xavier Sigaud 150, Rio de Janeiro 22290-180, Brazil; 2 La Plata National University and Argentina’s National Research Council (IFLP-CCT-CONICET)-C. C. 727, La Plata 1900, Argentina

**Keywords:** self-organized criticality, generalized entropies, nonextensive thermostatistics, information theory, 05.90.+m, 05.70.Ce, 05.40.Fb

## Abstract

Events occurring with a frequency described by power laws, within a certain range of validity, are very common in natural systems. In many of them, it is possible to associate an energy spectrum and one can show that these types of phenomena are intimately related to Tsallis entropy $S_{q}$. The relevant parameters become: (i) The entropic index *q*, which is directly related to the power of the corresponding distribution; (ii) The ground-state energy $\varepsilon_{0}$, in terms of which all energies are rescaled. One verifies that the corresponding processes take place at a temperature $T_{q}$ with $kT_{q}\propto \varepsilon_{0}$ (i.e., isothermal processes, for a given *q*), in analogy with those in the class of self-organized criticality, which are known to occur at fixed temperatures. Typical examples are analyzed, like earthquakes, avalanches, and forest fires, and in some of them, the entropic index *q* and value of $T_{q}$ are estimated. The knowledge of the associated entropic form opens the possibility for a deeper understanding of such phenomena, particularly by using information theory and optimization procedures.

## 1. Introduction

Power laws are ubiquitous in many areas of knowledge, emerging in economics, natural and social sciences, among others [1]. In the latest years, a particular interest has been given to frequency of events, which very often follow power laws: (i) In humanities, the Zipf’s law states that the relative word frequency in a given text is inversely proportional to its rank (defined as its position in a rank of decreasing frequency); (ii) In natural sciences, the frequency of earthquakes with a magnitude larger than a certain value *m*, plotted versus *m*, leads to the Gutenberg–Richter law [2]; furthermore, the frequency of avalanches, as well as of forest fires, of a given size *l*, plotted versus *l*, yield power laws [1]. Simple dynamic scale-free models, without tuning of a control parameter, but sharing many features of the critical point in a standard phase transition, like long-range correlations, have been introduced to approach theoretically the types of phenomena in examples (ii). For these reasons, the term self-organized criticality (SOC) [3] was coined, considered as the main characteristic exhibited by these models; since then, a vast literature appeared in this area (for reviews, see References [4,5,6,7]). Although stationary states may occur in SOC models, they are essentially characterized by out-of-equilibrium states, and in many cases jumps between different states occur due to energy changes; consequently, equilibrium thermodynamics does not apply to these models. Moreover, one of the most curious aspects concerns the fact that a critical state is approached without a temperature-like control parameter, and one of the most relevant questions concerns which real systems are well-described by SOC models, and under what conditions SOC applies [7].

Recently, a wide variety of entropic forms have been considered in the literature, either in the context of information theory, or for approaching real phenomena (see, e.g., References [8,9,10,11,12,13,14,15,16,17,18]). Many of these proposals recover the well-known Boltzmann–Gibbs entropy [19,20] as particular limits, and are usually known as generalized entropic forms. In the present work we show a connection between phenomena following power-law frequency of events and Tsallis $S_{q}$ entropy [9,10,11]. For this purpose, we develop a framework that could be relevant for some of the phenomena described in the previous paragraph. In this proposal we assume the existence of equilibrium (or long-living metastable) states, characterized by an energy spectrum $\left\{\epsilon_{i}\right\}$, which represent notorious differences with respect to the SOC models. The main motivation is that in many cases it is possible to define an energy-like variable, related in some way to one of the relevant parameters of the system, e.g., the magnitude of an earthquake, or the size of an avalanche, should be associated to some quantity of energy released. Since these parameters obey power laws, one expects that their corresponding energies should be also power-like distributed, leading an energy probability distribution $p\left(\epsilon \right)∼\epsilon^{-\gamma }$, where γ∈ℜ, restricted to γ>1, for reasons that will become clear later.

Then, from the distribution p(ϵ) we follow previous works, where a procedure for calculating fundamental quantities (like the partition function) was developed, by combining information theory and a key thermodynamical relation (see, e.g., References [21,22,23,24]). More precisely, we calculate the internal, or more generally, average energy *U* and define a general entropic form satisfying basic requirements [19,20], like being a functional that depends only on the set of probabilities. Furthermore, imposing the fundamental relation of thermodynamics,

(1) dU=TdS,

We obtain the associated entropy and verify that the temperature should be constant, for consistency. Curiously, the distribution p(ϵ) turns up to be temperature-independent, and consequently, all average values calculated from this probability distribution become independent of the temperature. Hence, similarly to what happens in SOC models, in the present approach the temperature does not play a crucial role for these types of phenomena.

In the next section we review some results of References [21,22,23,24], and especially how to combine general concepts of information theory with the fundamental relation of Equation (1), with the purpose of deriving an equation for obtaining the entropic form from a given energy spectrum. In Section 3 we discuss energy power-law distributions, and show a peculiar behavior, namely, that through the normalization procedure its dependence on the temperature disappears. Consequently, all quantities derived from these distributions, like average values, do not depend on the temperature. In Section 4 we analyze data of events within the present framework, by associating the corresponding power-law distributions with the energy distributions discussed in Section 3. Finally, in Section 5 we present our main conclusions.

## 2. Combining Information Theory and Thermodynamics

Herein we review some basic results of References [21,22,23,24], which were derived by considering a nondegenerate energy spectrum $\left\{\epsilon_{i}\right\}$. Hence, a discrete index *i* will identify uniquely a state with an energy $\epsilon_{i}$, occurring with a probability $p_{i}$, in such a way that the internal energy is defined as

(2) $$U=\sum_{i}\;\epsilon_{i}\;p_{i}\;.$$

Moreover, let $g(p_{i})$ be an arbitrary concave smooth function of $p_{i}$; we assume that the entropic functional may be written in the form [19,20]

(3) $$S\left(\left\{p_{i}\right\}\right)=k\sum_{i}g\left(p_{i}\right)\;\left[g\left(p_{i}\right)=0,\;\;\;\mathrm{if}\;\;\;p_{i}=0,\;\;\mathrm{or}\;\;\;p_{i}=1\right],$$

where *k* is a positive constant with entropy dimensions.

Let us now consider a small change in the level populations (which may occur, e.g., due to an infinitesimal exchange of heat); then, the probabilities $\left\{p_{i}\right\}$ will vary according to,

(4) $$p_{i}\to p_{i}\;+\;dp_{i},\;\mathrm{with}\;\sum_{i}\;dp_{i}=0\;,$$

with the last condition resulting from normalization ($\sum_{i}\;p_{i}=1$). This procedure will in turn generate infinitesimal changes in the entropy and internal energy, and we impose the fundamental relation of Equation (1). One obtains (up to first order in $dp_{i}$) [21],

(5) $$\sum_{i}\left[\epsilon_{i}-kTg^{\prime }\left(p_{i}\right)\right]dp_{i}\equiv \sum_{i}K_{i}dp_{i}=0,$$

where the prime indicates a derivative with respect to $p_{i}$. As shown in Reference [21], Equations (4) and (5) lead to just one expression for the $p_{i}$ and further, that all $K_{i}$ should be equal. The resulting value K is found through the normalization condition on the ensuing probability distribution (K is, in fact, related to the partition function), to be determined by the relation,

(6) $$K=\epsilon_{i}\;-kTg^{\prime }\left(p_{i}\right)\;\Rightarrow \;g^{\prime }\left(p_{i}\right)=\beta \left(\epsilon_{i}\;-K\right)\;;\;\;\left(\beta \equiv 1/kT\right)\;.$$

From now on we will consider, for simplicity, a continuous energy spectrum represented by an energy probability distribution p(ϵ), defined in a given range of energies between a minimum value $\epsilon_{0}$, and a maximum $\epsilon_{m}$. Although the events to be studied herein are expressed in terms of discrete sets of data, we will associate to them continuous distributions, which result from fittings of these data in such a range, as will be defined appropriately in the following sections. In the next section we define the probability distribution p(ϵ) of interest for the present work, and calculate relevant quantities; moreover, we consider the continuous form of Equation (6) to obtain the associated entropic form.

## 3. Power-Law Distributions and Associated Entropy

Power-law distributions frequently appear to be valid for certain ranges of its parameters, in variegated empirical settings pertaining to diverse disciplines [1,2,3,4,5,6,7]. We enlarge the scope of our methodology by considering systems for which a strict underlying thermodynamics does not exist, the inverse-temperature β being just a measure of the probability-distribution’s ”spread”. Let us then consider an energy spectrum following a power-law distribution, defined in a given range of energies between a minimum value $\epsilon_{0}$, and a maximum $\epsilon_{m}$,

(7) $$p\left(\epsilon \right)=\frac{1}{Z}\;{\left(\beta \epsilon \right)}^{-\gamma }\;\left(\gamma >1;\;\;\epsilon_{0}\leq \epsilon \leq \epsilon_{m}\right),$$

with a non-negative ground-state energy, $\epsilon_{0}\geq 0$. The normalization condition,

(8) $$\int_{\epsilon_{0}}^{\epsilon_{m}}d\epsilon \;p\left(\epsilon \right)=1\;,$$

yields

(9) $$Z=\int_{\epsilon_{0}}^{\epsilon_{m}}d\epsilon \;{\left(\beta \epsilon \right)}^{-\gamma }=\frac{\epsilon_{0}}{\gamma -1}\;{\left(\beta \epsilon_{0}\right)}^{-\gamma }\left[1-{\left(\frac{\epsilon_{0}}{\epsilon_{m}}\right)}^{\gamma -1}\right]\;,$$

leading to

(10) $$p\left(\epsilon \right)=\frac{\gamma -1}{\epsilon_{0}\left[1-{\left(\epsilon_{0}/\epsilon_{m}\right)}^{\gamma -1}\right]}{\left(\frac{\epsilon }{\epsilon_{0}}\right)}^{-\gamma }\;,$$

which does not depend upon β.

One should notice that, in order to obtain an appropriate power-law decay from the distribution above one should have γ∈ℜ, restricted to γ>1. Furthermore, p(ϵ) presents dimensions ${\left[\mathrm{energy}\right]}^{-1}$, as required by Equation (8).

One curious aspect of p(ϵ) in Equation (10) concerns its non-dependence on the parameter β, which, although introduced in Equation (7), it cancelled by imposing normalization; later on, it will be shown that the parameter β takes a constant value, for consistency. Consequently, all properties derived from the probability distribution of Equation (10) will not allow variations on the temperature; as an example, one has the average energy,

(11) $$U=\int_{\epsilon_{0}}^{\epsilon_{m}}d\epsilon \;\epsilon \;p\left(\epsilon \right)=\epsilon_{0}\;\frac{\left(\gamma -1\right)\left[1-{\left(\epsilon_{0}/\epsilon_{m}\right)}^{\gamma -2}\right]}{\left(\gamma -2\right)\left[1-{\left(\epsilon_{0}/\epsilon_{m}\right)}^{\gamma -1}\right]}\;.$$

As mentioned before, the present approach holds for any γ>1; the particular limit γ→2 of the internal energy above may be obtained through the l’Hopital rule,

(12) $$U=\lim_{\gamma \to 2}\;\epsilon_{0}\;\frac{\left(\gamma -1\right)\left[1-\exp\left(\gamma -2\right)\ln\left(\epsilon_{0}/\epsilon_{m}\right)\right]}{\left(\gamma -2\right)\left[1-{\left(\epsilon_{0}/\epsilon_{m}\right)}^{\gamma -1}\right]}=-\epsilon_{0}\;\frac{\ln\left(\epsilon_{0}/\epsilon_{m}\right)}{1-\left(\epsilon_{0}/\epsilon_{m}\right)}\;.$$

In order to deal appropriately with the continuous form of Equation (6), we define the dimensionless quantities,

(13) $$\tilde{p}\left(\tilde{\epsilon }\right)=p\left(\epsilon \right)\epsilon_{0}\;;\;\tilde{\epsilon }=\frac{\epsilon }{\epsilon_{0}}\;\left(\tilde{\epsilon }\geq 1\right)\;,$$

so that Equation (10) may be expressed as

(14) $$\tilde{p}\left(\tilde{\epsilon }\right)=\frac{\tilde{\epsilon }{\;}^{-\gamma }}{B}\;;\;\;B=\frac{1}{\gamma -1}\left[1-{\left(\frac{\epsilon_{0}}{\epsilon_{m}}\right)}^{\gamma -1}\right]=\frac{1}{\gamma -1}\left[1-{\left(\frac{1}{{\tilde{\epsilon }}_{m}}\right)}^{\gamma -1}\right]\;,$$

whereas the normalization condition becomes

(15) $$\int_{\epsilon_{0}}^{\epsilon_{m}}d\epsilon \;p\left(\epsilon \right)=\int_{1}^{{\tilde{\epsilon }}_{m}}d\tilde{\epsilon }\;\tilde{p}\left(\tilde{\epsilon }\right)=1\;.$$

The continuous form of Equation (6) becomes

(16) $$g^{\prime }\left(\tilde{p}\right)=\beta \left[\epsilon_{0}\tilde{\epsilon }\left(\tilde{p}\right)-K\right]\;,$$

and we are using the fact that $\tilde{p}\left(\tilde{\epsilon }\right)$ is of monotonic decreasing nature, so that it can be inverted, yielding a function $\tilde{\epsilon }\left(\tilde{p}\right)$. Notice that Equation (16) is a first-order differential equation for $g(\tilde{p})$, in fact a Bernoulli equation of zeroth-order; its solution reads,

(17) $$g\left(\tilde{p}\right)=\beta \int \;\left[\epsilon_{0}\tilde{\epsilon }\left(\tilde{p}\right)-K\right]\;d\tilde{p}\;+\;C^{\prime }\;.$$

Now, one can invert Equation (14), so that $\tilde{\epsilon }\left(\tilde{p}\right)=B^{-1/\gamma }\;{\tilde{p}}^{-1/\gamma }$, and substitute this result in Equation (17), leading to

(18) $$g\left[\tilde{p}\left(\tilde{\epsilon }\right)\right]=-\beta K\tilde{p}\left(\tilde{\epsilon }\right)+\beta \epsilon_{0}B^{-1/\gamma }\;\frac{{\left[\tilde{p}\left(\tilde{\epsilon }\right)\right]}^{1-1/\gamma }}{1-1/\gamma }+C^{\prime }\;.$$

Using the conditions of Equation (3), i.e., $g[\tilde{p}\left(\tilde{\epsilon }\right)]=0$, for $\tilde{p}\left(\tilde{\epsilon }\right)=0$ and $\tilde{p}\left(\tilde{\epsilon }\right)=1$, one obtains that $C^{\prime }=0$ and

(19) $$K=\frac{B^{-1/\gamma }}{1-1/\gamma }\;\epsilon_{0}\;,$$

showing that K is indeed related to the normalization of the probability distribution. Hence, Equation (18) becomes

(20) $$g\left[\tilde{p}\left(\tilde{\epsilon }\right)\right]=\frac{\beta \epsilon_{0}B^{-1/\gamma }}{1-1/\gamma }\left\{-\tilde{p}\left(\tilde{\epsilon }\right)+{\left[\tilde{p}\left(\tilde{\epsilon }\right)\right]}^{1-1/\gamma }\right\}\;,$$

leading to

(21) $$S\left[\tilde{p}\right]=k\int d\tilde{\epsilon }\;g\left[\tilde{p}\left(\tilde{\epsilon }\right)\right]=\frac{k\beta \epsilon_{0}B^{-1/\gamma }}{1-1/\gamma }\int d\tilde{\epsilon }\;\left\{-\tilde{p}\left(\tilde{\epsilon }\right)+{\left[\tilde{p}\left(\tilde{\epsilon }\right)\right]}^{1-1/\gamma }\right\}\;.$$

By recourse to the exact mapping detailed below, the expression above may be identified with Tsallis entropy [9,10,11],

(22) $$S_{q}\left[\tilde{p}\right]=\frac{k}{q-1}\left(1-\int d\tilde{\epsilon }\;\tilde{p}{\;}^{q}\right)\;,$$

through

(23) $$1-\frac{1}{\gamma }=q\;;\;\frac{\beta \epsilon_{0}B^{-1/\gamma }}{1-1/\gamma }=\frac{1}{1-q}\;,$$

where *q* represents the usual entropic index. This is of practical utility because we have now at our disposal the large set of useful recipes developed since 1988 with regards to Tsallis’ measure. Now, manipulating Equations (14) and (23), we obtain

(24) $$\beta \epsilon_{0}={\left(\frac{q}{1-q}\right)}^{q}{\left[1-{\left(\frac{\epsilon_{0}}{\epsilon_{m}}\right)}^{q/(1-q)}\right]}^{1-q}\;,$$

showing that the parameter β should assume a real constant value, for a given value of 0<q<1. Hence, defining a fixed pseudo-temperature $T_{q}$, such that the spread $\beta =1/(kT_{q})$, one finds

(25) $$kT_{q}=\epsilon_{0}{\left(\frac{1-q}{q}\right)}^{q}{\left[1-{\left(\frac{\epsilon_{0}}{\epsilon_{m}}\right)}^{q/(1-q)}\right]}^{q-1}\;.$$

In this way, the probability distribution of Equation (10), *which is indeed a power-law*, may be expressed in terms of the entropic index *q*,

(26) $$p\left(\epsilon \right)=\frac{q}{\left(1-q\right)\epsilon_{0}}\frac{1}{\left[1-{\left(\epsilon_{0}/\epsilon_{m}\right)}^{q/(1-q)}\right]}{\left(\frac{\epsilon_{0}}{\epsilon }\right)}^{1/(1-q)}\;,$$

being defined for 0<q<1 only; notice that this restriction is equivalent to γ>1 (cf. Equation (23)).

For several of the examples to be considered below, the associated energy spectra will be characterized by $\epsilon_{m}\gg \epsilon_{0}$, so the Equation (25) may be expanded in a power series, e.g.,

(27) $$kT_{q}=\epsilon_{0}{\left(\frac{1-q}{q}\right)}^{q}\left[1+\left(1-q\right){\left(\frac{\epsilon_{0}}{\epsilon_{m}}\right)}^{q/(1-q)}+\cdots \right]\;,$$

whereas for the probability distribution one has the approximate expression

(28) $$p\left(\epsilon \right)=\frac{q}{\left(1-q\right)\epsilon_{0}}{\left(\frac{\epsilon_{0}}{\epsilon }\right)}^{1/(1-q)}\left[1+{\left(\frac{\epsilon_{0}}{\epsilon_{m}}\right)}^{q/(1-q)}+\cdots \right]\;,$$

which is not a *q*-exponential.

The expansions of Equations (27) and (28) show that the maximum energy value $\epsilon_{m}$ only appears in higher-order corrections of $T_{q}$ and p(ϵ). In such cases, the most relevant parameters in Equation (10) become the exponent γ [directly related to *q* through Equation (23)] and the ground-state energy $\epsilon_{0}$.

One should focus attention upon the curious result we have obtained in this effort. We were able to relate with Tsallis entropy the power-law distribution Equation (7) (not the usual *q*-exponential distribution). In fact, the equilibrium distribution that arises out of the extremization procedure for a given entropic form depends directly on the constraints imposed and the choices made regarding the corresponding Lagrange multipliers [10]. As shown in Reference [24], the distribution Equation (7) may be obtained from an extremization procedure effected on Tsallis entropy in Equation (22), by considering the usual constraints of probability normalization (associated Lagrange multiplier $\tilde{\alpha }$) and internal energy definition in Equation (2) (corresponding Lagrange multiplier β), by choosing appropriately the first Lagrange multiplier, i.e.,

(29) $$\tilde{\alpha }=-\frac{Z^{-1/\gamma }}{1-1/\gamma }\;.$$

In the following section we will analyze examples of real systems governed by a power-law frequency of events.

## 4. Typical Examples in Natural Systems: From Data of Events to Energy Spectrum

Next, we describe some examples, chosen from the literature, of power-law distributions found in natural systems. In order to associate these examples with the theoretical approach of the previous sections, we will assume that: (i) The relevant variable of each distribution may be related in some way to the energy ϵ; (ii) The fittings describing each class of phenomena may be associated with the continuous probability distribution of Equation (10), defined in the range between its minimum and maximum values ($\epsilon_{0}$ and $\epsilon_{m}$, respectively). We discuss separately two types of phenomena: (i) Systems presenting energy power-law distributions that can be directly related to the distribution of Equation (10). In such cases, we calculate, from the corresponding data, important quantities like the entropic index *q*, the dimensionless ratio $\epsilon_{0}/\epsilon_{m}$, and the fixed value of the temperature $T_{q}$; (ii) Systems presenting power-law distributions P(x), depending on a parameter *x* that can be related to the energy ϵ through some invertible monotonic function. For these cases, we propose a procedure for calculating the quantities of interest.

### 4.1. Systems Exhibiting Energy Power-Law Distributions

Certainly, one of the most paradigmatic power-law distributions is the Gutenberg–Richter law, which measures the frequency of earthquakes with a magnitude larger than a certain value *m* [2]. The magnitude *m* may be related to the seismic energy (or energy released) *E* [25], so that the Gutenberg–Richter law is sometimes expressed in a form similar to Equation (10),

(30) $$p\left(E\right)∼E^{-\gamma^{\prime }}\;.$$

In fact, as pointed out in Reference [26], the distribution above was proposed previously by Wadati (1932) in a paper written in japonese [27]. By analyzing earthquakes around the Tokyo station, Wadati obtained two different estimates for the exponent $\gamma^{\prime }$, respectively $\gamma^{\prime }=1.7$ and $\gamma^{\prime }=2.1$, under different assumptions for the distributions of hypocenters. One should notice that the first estimate is very close to $\gamma^{\prime }=5/3$, which is nowadays generally accepted for the index of the power-law distribution of seismic energies [26,28]. For earthquakes, one can assume that the seismic energy *E* can be related to the energy ϵ in a simple way, e.g., at most, apart from a proportionality constant, ϵ∝E, so that Equation (30) can be associated with the probability distribution of Equation (10). Under this assumption one has $\gamma^{\prime }=\gamma$, and using Equation (23) one obtains the entropic index q=2/5 for earthquakes.

Recently, the possibility of investigating seismic phenomena by means of laboratory experiments has gained a big motivation after the identification of deep associations between earthquakes and the fracture of materials [29,30]. As examples, one may mention experiments of compression on porous glasses [31,32], as well as on small wood samples [33]. This connection is based on the crackling noise idea, where systems under slow perturbations may respond through discrete events covering a wide variety of amplitudes. By recording the amplitudes of these cracking noises, one can compute the associated energies, which may be normalized conveniently in such a way to produce energy probability distributions. Inspired by those, further experiments have been carried out by considering different apparatus, e.g., without compression, through the analysis of the acoustic emission in a variety of systems, like crumpled plastic sheets [34], or ethanol-dampened charcoal [35].

**Figure 1:**
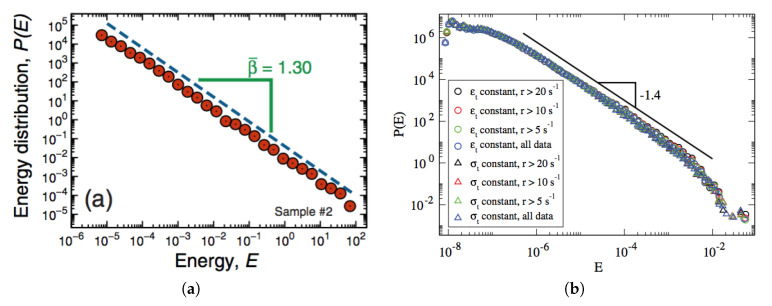
Typical energy power-law distributions found in experiments. (**a**) Energy distribution P(E) versus *E*, obtained from the cracking noise produced by charcoal samples, when dampened with ethanol (from Reference [35]). (**b**) Energy distribution P(E) versus *E*, obtained from acoustic emission in small wood samples under compression. Data from different experimental conditions, i.e., constant strain rate $\varepsilon_{t}$, constant stress rate $\sigma_{t}$, and various event rates r(t) (defined as the number of events in a time interval divided by the interval length), all fall in a universal probability distribution (from Reference [33]). In both cases, the variable *E* is properly normalized and defined as a dimensionless quantity; within the present approach (cf. Equation (10)), these examples correspond to γ=1.3 (case (a)) and γ=1.4 (case (b)).

The two examples presented in Figure 1 follow these procedures, where the energy probability distribution P(E) is represented versus *E* for two distinct experiments. Results from the cracking noise produced by charcoal samples, when dampened with ethanol, are presented in Figure 1a; through their experiments, the authors have shown that the most fundamental seismic laws ruling earthquakes could be reproduced [35]. In an analogous way, avalanches were observed recently by means of acoustic emission in small wood samples under compression; these avalanches show results very similar to earthquakes and crackling noise in rocks and laboratory tests on brittle materials [33]. The distributions of energies are shown in Figure 1b, where data from different experimental conditions, i.e., constant strain rate $\varepsilon_{t}$, constant stress rate $\sigma_{t}$, and distinct event rates r(t) (defined as the number of events in a time interval divided by the interval length), all fall in a universal probability distribution P(E). Like done before for natural earthquakes, in both cases one can identify directly the energy liberated *E* with ϵ, i.e., ϵ∝E, so that the probability distribution of Equation (10) can be related with the fitting distributions P(E) shown in Figure 1a,b. In this way, these examples correspond respectively, to γ=1.3 and γ=1.4, representing smaller values when compared to γ=5/3 generally accepted for earthquakes. From Equation (23) one obtains the entropic indexes q≈0.23 (Figure 1a) and q≈0.29 (Figure 1b). Moreover, in the plots of Figure 1 one has very small values for $\epsilon_{0}/\epsilon_{m}$ (typically, $\left(\epsilon_{0}/\epsilon_{m}\right)<10^{-4}$), so that the expansions of Equations (27) and (28) are well approximated by their leading-order contributions. In particular, the dimensionless temperature of Equation (27) becomes $\left(kT_{q}/\epsilon_{0}\right)\approx {\left[\left(1-q\right)/q\right]}^{q}$, so that the two examples of Figure 1 can be associated with fixed values of the dimensionless temperature, $(kT_{q}/\epsilon_{0})\approx 1.32$ (Figure 1a) and $(kT_{q}/\epsilon_{0})\approx 1.30$ (Figure 1b). One notices that the estimates of *q* and $T_{q}$ are very close to one another in these two experiments.

### 4.2. Systems Exhibiting General Power-Law Distributions: Identifying Relevant Variables with Energy

**Figure 2:**
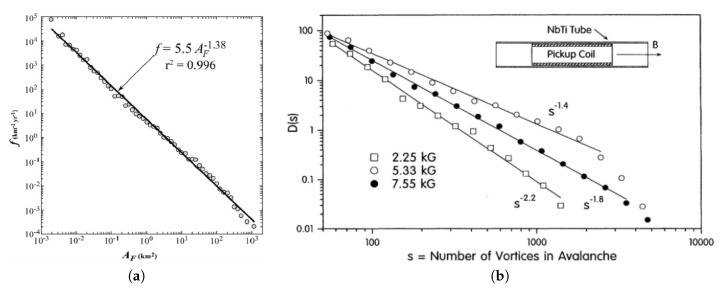
Typical power-law distributions found in natural systems. (**a**) Forest-fire frequency density per year is represented versus forest burned area $A_{F}$; the data corresponds to the period 1976–1996 in Ontario, Canada (from Reference [36]). (**b**) Probability density for measuring an avalanche of *s* vortices [D(s)] in a hard superconductor is represented versus *s*, for three different values of the magnetic field. The inset shows a sketch of the experimental arrangement, where one has a tubular NbTi sample and the pickup coil. Large variations of the voltage measured in the pickup coil are associated with avalanches (from Reference [37]).

Let us now analyze systems characterized by a given parameter *x* and its associated power-law distribution P(x); contrary to the examples shown in Figure 1, the relation between *x* and ϵ does not follow straightforwardly; two typical examples in this class are shown in Figure 2. In Figure 2a the forest-fire frequency density per year is represented versus forest burned area $A_{F}$. The straight line yields a frequency versus area power-law distribution with an exponent 1.38; the data corresponds to Ontario, Canada, during the period 1976–1996 [36]. Results from experiments carried out on a NbTi (conventional superconductor) sample, at the Bean critical state, are exhibited in Figure 2b [37]. For hard superconductors, the Bean critical state corresponds to a marginal stable state, where the Lorentz force acting on each vortex equals the maximum pinning force. A sketch of the experimental arrangement is represented in the inset, where one has a tubular NbTi sample and the pickup coil. An external magnetic field enters the interior of the tube, inducing a voltage on the pickup coil; large variations of the voltage in the pickup coil are associated with avalanches. The corresponding probability density for measuring an avalanche of *s* vortices is represented versus *s* (cf. Figure 2b), for three different values of the magnetic field (the exponent of the power-law distribution is field-dependent); one notices that for the higher value of the magnetic field (7.55 kG), one gets avalanches up to 5000 vortices. In both examples shown in Figure 2, one expects the variable ϵ of the previous section to be an increasing function of the relevant variable, i.e., of the burned area $A_{F}$ (Figure 2a), as well as of the energy required for producing *s* vortices in a given avalanche (Figure 2b).

In order to relate probability distributions associated to these types of events to the approach of the previous sections, let us consider a given set of discrete data $\left\{x_{i}\right\}$, given by m+1 values $\left(x_{0},x_{1},x_{2},\cdots ,x_{m}\right)$, ordered in such a way that $0\leq x_{0}\leq x_{1}\leq x_{2}\cdots x_{m-1}\leq x_{m}$. Moreover, each quantity $x_{i}$ occurs with a frequency $c_{i}$, following

(31) $$\sum_{i=0}^{m}c_{i}=C\;;\;c_{0}\geq c_{1}\geq c_{2}\cdots c_{m-1}\geq c_{m}\;.$$

Rescaling the set of variables by its minimum value $x_{0}$, one gets a discrete set of dimensionless data $\left\{{\tilde{x}}_{i}\right\}$, $1\leq {\tilde{x}}_{1}\leq {\tilde{x}}_{2}\cdots {\tilde{x}}_{m-1}\leq {\tilde{x}}_{m}$, each ${\tilde{x}}_{i}$ occurring with a probability $P_{i}\left({\tilde{x}}_{i}\right)$ [$P_{i}\left({\tilde{x}}_{i}\right)=c_{i}/C$ (i=0,1,2,⋯,m) representing a set of decreasing probabilities], so that

(32) $$\sum_{i=0}^{m}P_{i}\left({\tilde{x}}_{i}\right)=1\;.$$

Herein we will be interested in the kind of phenomena illustrated in Figure 2, which are well-fitted by continuous power-law distributions; furthermore, we define dimensionless quantities similarly to those of Equations (13) and (14), i.e.,

(33) $$\tilde{P}\left(\tilde{x}\right)=\frac{1}{A}\;{\tilde{x}}^{-\alpha }\;\left(\alpha >1;\;\;1\leq \tilde{x}\leq {\tilde{x}}_{m}\right),$$

where now $\tilde{x}$ corresponds to the continuous representation of the discrete variables $\left\{{\tilde{x}}_{i}\right\}$, whereas $\tilde{P}\left(\tilde{x}\right)$ denotes a dimensionless probability distribution. Moreover, the normalization condition,

(34) $$\int_{1}^{{\tilde{x}}_{m}}d\tilde{x}\;\tilde{P}\left(\tilde{x}\right)=1\;,$$

requires

(35) $$A=\frac{1}{\alpha -1}\left[1-{\left(\frac{x_{0}}{x_{m}}\right)}^{\alpha -1}\right]=\frac{1}{\alpha -1}\left[1-{\left({\tilde{x}}_{m}\right)}^{1-\alpha }\right]\;.$$

Accordingly, one can also calculate the average value,

(36) $$\langle \tilde{x}\rangle =\int_{1}^{{\tilde{x}}_{m}}d\tilde{x}\;\tilde{P}\left(\tilde{x}\right)\tilde{x}=\frac{\left(\alpha -1\right)\left[1-{\left({\tilde{x}}_{m}\right)}^{2-\alpha }\right]}{\left(\alpha -2\right)\left[1-{\left({\tilde{x}}_{m}\right)}^{1-\alpha }\right]}\;.$$

One should notice the resemblance of the probability distribution $\tilde{P}\left(\tilde{x}\right)$ of Equation (33) with the energy distribution of $\tilde{p}\left(\tilde{\epsilon }\right)$ in Equation (14), as well as of the average value $\langle \tilde{x}\rangle$ with the internal energy of Equation (11). Such similarities suggest that $\tilde{\epsilon }$ and $\tilde{x}$ should be directly related to one another; herein, we propose

(37) $$\tilde{\epsilon }=\Lambda \left(\tilde{x}\right)\;\Rightarrow \;\epsilon =\epsilon_{0}\;\Lambda \left(\frac{x}{x_{0}}\right)\;,$$

where Λ(y) represents an invertible and monotonically increasing function of *y*, such that Λ(1)=1. The normalization condition on both distributions $\tilde{P}\left(\tilde{x}\right)$ and $\tilde{p}\left(\tilde{\epsilon }\right)$ requires that

(38) $$\tilde{P}\left(\tilde{x}\right)d\tilde{x}=\tilde{p}\left(\tilde{\epsilon }\right)d\tilde{\epsilon }\;;\;\Rightarrow \;\tilde{P}\left(\tilde{x}\right)\left|\frac{d\tilde{x}}{d\tilde{\epsilon }}\right|=\tilde{p}\left(\tilde{\epsilon }\right)\;,$$

which implies that $\tilde{\epsilon }$ and $\tilde{x}$ should be related through a power, i.e., $\Lambda \left(y\right)=y^{\nu }$, with ν being a positive real number. In this way, one obtains the relation between the two variables,

(39) $$\tilde{\epsilon }={\tilde{x}}^{\nu }\;.$$

Therefore, the internal energy of Equation (11) may be written as

(40) $$U=\epsilon_{0}\;\langle \tilde{\epsilon }\rangle =\epsilon_{0}\;\langle {\tilde{x}}^{\nu }\rangle =\epsilon_{0}\;\int_{1}^{{\tilde{x}}_{m}}d\tilde{x}\;{\tilde{x}}^{\nu }\;\tilde{P}\left(\tilde{x}\right)=\frac{\epsilon_{0}\left(\alpha -1\right)}{\alpha -1-\nu }\;\frac{1-{\tilde{x}}_{m}^{\nu }{\tilde{x}}_{m}^{1-\alpha }}{1-{\tilde{x}}_{m}^{1-\alpha }}\;,$$

which recovers the result of Equation (11) by using ${\tilde{\epsilon }}_{m}={\tilde{x}}_{m}^{\nu }$ and imposing the relation

(41) $$\frac{\alpha -1}{\nu }=\gamma -1\;.$$

Hence, for systems exhibiting power-law distributions presenting a dependence on a general parameter *x*, being characterized by an exponent α according to Equation (33), the entropic form of Equation (22) still applies. In order to identify the entropic index *q*, one should carry out the following procedure: (i) Obtain the exponent ν relating the energy ϵ to the relevant parameter *x* through Equation (39); (ii) The exponent α is taken directly from the data, like those in Figure 2, e.g., α=1.38 in the case of forest fires (Figure 2a). Then, use Equation (41) to calculate the exponent γ of the corresponding energy distribution; (iii) Calculate the entropic index *q* by means of Equation (23). In many cases step (i) may become the most difficult task, since obtaining an energy distribution from a given set of data of natural systems may not be so obvious.

## 5. Conclusions

We have analyzed events that occur with a frequency following power laws, within a certain range of validity of their relevant parameters. These types of phenomena are very common in natural systems and are usually associated with self-organized criticality. In many of such cases it is possible to introduce an energy spectrum, defined in a given interval of energies between a minimum value $\epsilon_{0}$, and a maximum $\epsilon_{m}$, so that an internal energy may be calculated. Based on this, we have assumed the validity of the fundamental relation dU=TdS, and have calculated important quantities, like the associated entropic form and temperature. As a curious aspect, the power-law probability distribution is temperature-independent, in agreement with self-organized-criticality; however, we have shown that these phenomena occur at a constant temperature and follow Tsallis entropy $S_{q}$, with an entropic index 0<q<1; from the thermodynamical point of view, these phenomena could be identified as isothermal processes. In cases where $(\epsilon_{m}/\epsilon_{0})\gg 1$, the relevant parameters within this procedure become the entropic index *q*, which is directly related to the power of the corresponding distribution, and the ground-state energy $\varepsilon_{0}$, in terms of which all energies are rescaled. In particular, the corresponding processes take place at a temperature $T_{q}$ with $\left(kT_{q}/\epsilon_{0}\right)\approx {\left[\left(1-q\right)/q\right]}^{q}$.

Typical examples were analyzed, like earthquakes, avalanches, and forest fires, and in some of them, the entropic index *q* and value of $T_{q}$ were estimated. Specially for earthquakes, we obtained q=2/5 and $(kT_{q}/\epsilon_{0})\approx 1.18$. It should be mentioned that an analysis of probability distributions of energy differences (returns) of data from the Northern California earthquake catalogue has led to *q*-Gaussian distributions with q=1.75±0.15 [38]. Although the power-law distributions considered herein are very different from the *q*-Gaussian distribution of Reference [38], both are associated in some way to Tsallis entropy $S_{q}$; curiously, our estimate for the entropic index *q* agrees, within the error bars, with the result of Reference [38] by considering the usual correspondence q↔2−q.

The main contribution of the present work concerns the association of events occurring with a frequency following power laws with the entropy $S_{q}$, and that distinct types of events should be characterized by different values of *q*. Furthermore, the identification of an associated entropic form opens the possibility for a deeper understanding of such important natural phenomena, particularly by using information theory and optimization procedures.
